# Advanced Molecular and Immunological Diagnostic Methods to Detect SARS-CoV-2 Infection

**DOI:** 10.3390/microorganisms10061193

**Published:** 2022-06-10

**Authors:** John Charles Rotondo, Fernanda Martini, Martina Maritati, Elisabetta Caselli, Carla Enrica Gallenga, Matteo Guarino, Roberto De Giorgio, Chiara Mazziotta, Maria Letizia Tramarin, Giada Badiale, Mauro Tognon, Carlo Contini

**Affiliations:** 1 Department of Medical Sciences, University of Ferrara, 44121 Ferrara, Italy; mrf@unife.it (F.M.); mrtmtn@unife.it (M.M.); gllcln@unife.it (C.E.G.); mzzchr@unife.it (C.M.); marialetizi.tramarin@edu.unife.it (M.L.T.); giada.badiale@unife.it (G.B.); tgm@unife.it (M.T.); 2 Center for Studies on Gender Medicine, Department of Medical Sciences, University of Ferrara, 44121 Ferrara, Italy; 3 Laboratory for Technologies of Advanced Therapies (LTTA), University of Ferrara, 44121 Ferrara, Italy; 4 Orthopaedic Ward, Casa di Cura Santa Maria Maddalena, 45030 Occhiobello, Italy; 5 Section of Microbiology, CIAS Research Center and LTTA, Department of Chemical, Pharmaceutical and Agricultural Sciences, University of Ferrara, 44121 Ferrara, Italy; elisabetta.caselli@unife.it; 6 Department of Translational Medicine, St. Anna University Hospital of Ferrara, University of Ferrara, 44124 Ferrara, Italy; grnmtt@unife.it (M.G.); roberto.degiorgio@unife.it (R.D.G.)

**Keywords:** SARS-CoV-2, COVID-19, spike protein, RT-PCR, antigen-based immunoassays, ELISA, anti-SARS-CoV-2 antibodies, secretory IgA

## Abstract

**Simple Summary:**

COVID-19 emerged in late 2019 in China and rapidly spread across the globe. After 2 years, numerous advances have been made. First of all, the preventive vaccine, which has been implemented in record time, is effective in more than 95% of cases. Additionally, in the diagnostic field, there are numerous molecular and antigenic diagnostic kits available that are equipped with high sensitivity and specificity. Real Time-PCR-based assays for the detection of viral RNA are currently considered the gold-standard method for SARS-CoV-2 diagnosis while they can be used efficiently on pooled nasopharyngeal, or oropharyngeal samples for widespread screening. Moreover, additional, and more advanced molecular methods such as droplet-digital PCR (ddPCR), clustered regularly interspaced short palindromic repeats (CRISPR) and next-generation sequencing (NGS), are currently under development to detect SARS-CoV-2 RNA. However, as the number of subjects infected with SARS-CoV-2 is continuously increasing globally, health care systems are being placed under increased stress. Recent diagnostic strategies have been adopted to either detect viral antigens, i.e., antigen-based immunoassays, or human anti-SARS-CoV-2 antibodies, i.e., antibody-based immunoassays, in nasal or oropharyngeal swabs, as well as in blood or saliva samples. However, the role of mucosal sIgAs, which are essential in the control of viruses entering the body through mucosal surfaces, remains to be elucidated, and in particular the role of immune responses in counteracting SARS-CoV-2 infection, primarily at the site(s) of virus entry.

**Abstract:**

COVID-19 emerged in late 2019 in China and quickly spread across the globe, causing over 521 million cases of infection and 6.26 million deaths to date. After 2 years, numerous advances have been made. First of all, the preventive vaccine, which has been implemented in record time, is effective in more than 95% of cases. Additionally, in the diagnostic field, there are numerous molecular and antigenic diagnostic kits that are equipped with high sensitivity and specificity. Real Time-PCR-based assays for the detection of viral RNA are currently considered the gold-standard method for SARS-CoV-2 diagnosis and can be used efficiently on pooled nasopharyngeal, or oropharyngeal samples for widespread screening. Moreover, additional, and more advanced molecular methods such as droplet-digital PCR (ddPCR), clustered regularly interspaced short palindromic repeats (CRISPR) and next-generation sequencing (NGS), are currently under development to detect the SARS-CoV-2 RNA. However, as the number of subjects infected with SARS-CoV-2 continuously increases globally, health care systems are being placed under increased stress. Thus, the clinical laboratory plays an important role, helping to select especially asymptomatic individuals who are actively carrying the live replicating virus, with fast and non-invasive molecular technologies. Recent diagnostic strategies, other than molecular methods, have been adopted to either detect viral antigens, i.e., antigen-based immunoassays, or human anti-SARS-CoV-2 antibodies, i.e., antibody-based immunoassays, in nasal or oropharyngeal swabs, as well as in blood or saliva samples. However, the role of mucosal sIgAs, which are essential in the control of viruses entering the body through mucosal surfaces, remains to be elucidated, and in particular the role of the immune response in counteracting SARS-CoV-2 infection, primarily at the site(s) of virus entry that appears to be promising.

## 1. Introduction

SARS-CoV-2, which causes COVID-19, is the third coronavirus epidemic after the SARS-CoV-1 and MERS coronavirus outbreaks [1]. According to the European Centre for Disease Prevention and Control (ECDC), updated on 5 may 2022 [2], SARS-CoV-2 variants can be divided into the following three categories according to their pathogenic potential: (i) Variants of Concern (VOC), such as Delta and Omicron (BA.1 and BA.2 sublineages); (ii) variants of Interest (VOI), such as Omicron (BA.4 and BA.5 sublineages), and (iii) variants under monitoring (VUM), such as Omicron (BA.3 and BA.2 + L42X sublineages) [2].

SARS-CoV-2 infects host cells through ACE2 and TMPRSS2 receptors distributed in a wide variety of tissues [3] leading to a number of severe pathologies including pneumonia, acute myocardial injury, chronic damage to the cerebral system and other disorders that can affect the entire body [4]. The transmission of COVID-19 is primarily caused by droplets containing the virus generated by symptomatic patients [5], as well as probable asymptomatic subjects during disease incubation, although for the latter, there are no certain data available yet [6,7]. Touching an infected surface and then touching the eyes, nose and mouth without washing the hands can be a source of virus transmission.

Having infected and causing the death of thousands of individuals in China [8], the virus spread worldwide, reaching European countries including Italy [9,10] and the USA [11], with the number of confirmed new cases increasing every day. The spread of COVID-19 across the globe has caused over 521 million cases and 6.26 million deaths to date. Since the beginning of the pandemic, we learned that the clinical features of COVID-19 range from asymptomatic condition to severe/fatal lung injury and multi-organ failure due to an excessive immune response. Symptoms related to COVID-19 can be unspecific, but often include fatigue, fever and dry cough [12]. Unusual symptoms include nasal congestion, gastrointestinal discomfort, headache, conjunctivitis, pains and aches, as well as loss of taste and smell, lymphopenia, and prolonged symptomatic experience with antimicrobial therapy (at least 3 days) [6,13]. COVID-19 has also been associated with coagulation disorders, secondary bacterial infections, immunodeficiency and myocardial as well as hepatic, and renal injury [14,15]. The spectrum of symptoms is currently widening due to the identification of other effects of the disease, such as Long-COVID and Multisystem inflammatory syndrome in children (MIS-C), a rare and severe complication of SARS-CoV-2 infection that can affect the paediatric population [16,17].

These various clinical manifestations might depend on the patient’s SARS-CoV-2 load, pre- and co-existing pathologies, as well as factors such as age, gender, and genetics of the immune system, i.e., the HLA system. In fact, in severe cases, the mean viral load is 60 times higher than in mild cases. SARS-CoV-2 can infect many tissues and can be isolated from various biological fluids, which can be tested for the diagnosis of COVID-19 [1].

Although most infected individuals, especially young people under 30 years of age, do not require intervention or hospitalization, approximately one in five patients develop severe symptoms, such as difficult breathing [18,19]. Comorbid individuals, such as those with hypertension or heart and lung disease, diabetes, neoplasms, or generally those who are immunocompromised, are at increased risk of developing complications associated with SARS-CoV-2 infection [20].

With the high number of infections and deaths found so far worldwide, prevention, diagnosis and treatment of the disease and its complications along with vaccine research and development are more important than ever [12,21,22,23,24,25]. Because the COVID-19 pandemic has still not been brought under control and because it continues to grow rapidly around the world, it is imperative to achieve rapid diagnosis, especially in the early stages of the disease. 

Although SARS-CoV-2 detection is becoming more accurate via the refinement of molecular biology techniques [26], there are still a number of issues to be clarified. First, the exact determination of the viral load in asymptomatic subjects is necessary, i.e., healthy virus carriers, who theoretically carry a low viral load and low probability of infection and who will presumably continue to do so, and in symptom free individuals, who after a few days (1 to 3 days) will develop symptoms, becoming pre-symptomatic. Could they have higher viral load and thus be more likely to be infectious? Additionally, how long does this potential contagiousness last? Second, continued viral variants generated by people from continents where vaccination programs are limited, can often lead to difficulties in identification and negatively affect the current vaccination campaign [1,27]; the latest SARS-CoV-2 strains highlight the crucial role of the rapid and constant surveillance of the circulation of the SARS-CoV-2 variants including VOI and VUM Omicron sublineages, which are able to spread intensively, although remain less aggressive at the lung level [28]. Third, there is a lack of information regarding the detection of SARS-CoV-2 or its RNA in the main infection sites (eye) as well as in other viral targets expressing the ACE2 receptor [29,30]. The exact determination of viral loads at the eye level and of the local immune response against SARS-CoV-2, could instead be helpful. In particular, the anti-SARS-CoV-2 specific IgA determination in conjunctival fluid could represent a further step towards predicting lung mucosal invasion even in patients without ocular symptoms, as previously shown [14,31,32]. 

A nasopharyngeal swab is necessary to assess the presence/absence of genetic material of the pathogen, but this test does not discriminate between genomic molecules, which are much more numerous, and sub-genomic transcripts essential for the construction of new viral particles. SARS-CoV-2 could therefore be inactive, thwarted by our immune system, or active and effectively replicating [33]. 

A multitude of methods have been applied, demonstrating different areas of expertise in clinical, biomolecular, and mathematical-statistical fields can converge on a common ground. From real-time reverse transcription-polymerase chain reaction (RT-PCR) methods to the latest frontiers of clustered regularly interspaced short palindromic repeats (CRISPR)/Cas-based systems and artificial intelligence, much effort is being made to ensure not only the fast and safe identification of patients with SARS-CoV-2, but more importantly, that their prognosis can be improved with increasingly appropriate treatment protocols. The focus of this review article is diagnostic approaches for COVID-19 and its associated complications. The rapid growth in the daily rate of SARS-CoV-2 infection and the lack of suitable diagnostic tools for the early diagnosis of the virus represent important concerns for controlling the disease and preventing further the virus spread among people. The necessity for early and inexpensive identification even in remote areas of the globe with an effective result is urgently required. This early diagnostic approach can be performed using the biosensor-based tool.

## 2. Molecular Diagnostic Tools for SARS-CoV-2 Infection

**Figure 1:**
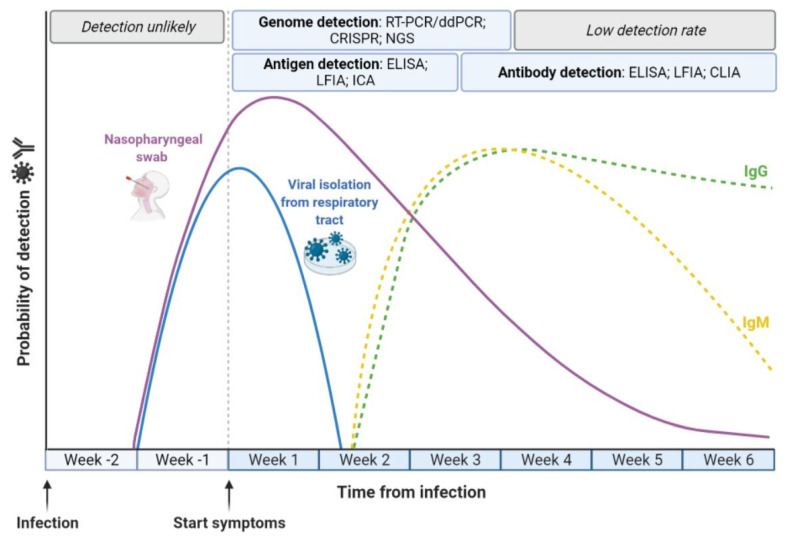
Progression of SARS-CoV-2 infection and in vitro diagnostics. The detection of active SARS-CoV-2 infection is performed using molecular diagnostics, i.e., real-time reverse transcription-polymerase chain reaction (RT-PCR), droplet-digital PCR (ddPCR), clustered regularly interspaced short palindromic repeats (CRISPR) and next-generation sequencing (NGS), in order to detect the viral RNA during the first weeks after the emergence of symptoms. In this period, it is also possible to detect the presence of SARS-CoV-2 components using antigen-based immunoassays, i.e., the enzyme-linked immunosorbent assay (ELISA), lateral flow immunoassay (LFIA) and immunochromatographic assay (ICA). Detection of SARS-CoV-2 antibodies (IgG and IgM) occurs through antibody-based immunoassays, i.e., ELISA, LFIA and chemiluminescence immunoassay (CLIA), from the third week after symptoms.

The pivotal strategy for COVID-19 management is the early, reliable and rapid identification SARS-CoV-2 infection (Figure 1) [34]. With the continuous development of numerous reliable molecular biology protocols and methods for both research and clinical purposes [35,36,37,38], the identification of SARS-CoV-2 has become highly sensitive and accurate [26,39]. Nucleic acid amplification is the most broadly used methodological approach for the identification and quantification of a large variety of pathogens [40,41], including viruses [42,43]. Overall, these methods comprise a variety of nucleic acid amplification-based tests, which have clearly demonstrated high reliability for the identification of previous pandemic caused by CoVs, including SARS-CoV-1 and MERS [44].

### 2.1. Real-Time Reverse Transcription-Polymerase Chain Reaction

The currently employed gold standard and most broadly used molecular method worldwide for the detection of active SARS-CoV-2 infection is RT-PCR [26,45,46]. The RT-PCR assay is a broadly employed molecular biology technique which allows for the analysis and quantification of nucleic acids for a large variety of applications [47,48,49]. The method is performed on nasopharyngeal and oropharyngeal swabs, which are the preferred sampling method of the World Health Organization (WHO) and the Centers for Disease Control and Prevention (CDC), although it can be underlined that oral throat wash (OTW) is an additional and less invasive, safer as well as more efficient sample-collection approach. Moreover, at the beginning of the pandemic, the WHO also presented a detailed list of PCR-based methods, protocols, safety information and limitations, from different international institutions for the detection of SARS-CoV-2 nucleic acid [50].

Overall, RT-PCR methods convert the initial RNA into complementary DNA (cDNA) by using an enzyme with reverse transcription activity. Then, cDNA is PCR amplified during a consecutive a series of thermal cycles (Ct; usually 35–40). Specifically designed gene-specific primers and labeled probes allow for the amplification of the cDNA [51,52]. The entire procedure requires 2–4 h to complete, according to the type of RT-PCR protocol employed. These characteristics confer a broad application potential for both research and clinical purposes to RT-PCR.

RT-PCR was the first methodological approach used for SARS-CoV-2 detection [46]. Since different CoVs are highly similar in terms of their genomic sequence, the analytical accuracy of SARS-CoV-2-based RT-PCR methods depends on the primer design for detecting various portions of the viral genome [53]. Protocols have been developed to amplify a variety of regions of the SARS-CoV-2 genome such as the spike (S), nucleocapsid (N), transmembrane (M), and envelope (E) genes, as well as the RNA-dependent RNA polymerase (RdRp), the open reading frame 1a (ORF1a), and ORF1b regions. A typical SARS-CoV-2-based RT-PCR assay is highly sensitive, although presents a limit of detection in the range of 0.3–100 copies/μL, which typically depends on the diagnostic method employed [54,55,56,57].

Since the beginning of the pandemic, hundreds of RT-PCR-based protocols/kits have been officially approved by several agencies, such as the Food and Drugs Administration (FDA) and the European Medicines Agency (EMA) [39,58,59]. Overall, these approved tests are able to detect two or three fragment regions of the SARS-CoV-2 genome, mainly using multiplex approaches based on standard RT-PCR procedures [59]. In general, RT-PCR kits have been optimized for detecting SARS-CoV-2 genomic sequences from several specimens including oropharyngeal nasopharyngeal, or nasal swabs, bronchoalveolar lavage, upper/lower respiratory tract aspirates as well as the sputum [57]. These kits mainly comprise various enzymes with reverse transcription and amplification activities, from two to three primers and probes sets, as well as negative/positive, internal controls.

The major advantage of RT-PCR-based methods compared to immunological tests is specificity and sensitivity. Regarding the limitations, several drawbacks have been identified over the years, from procedural contaminations to low sensitivity, thus leading in both cases to false/-positive and/or -negative results [57]. Thus, improving test specificity and sensitivity, while reducing the risk of false positive/negative results remains an unmet need [60]. It should be underlined that an additional limitation is that PCR-based methods are more expensive and less rapid than immunoassays such as rapid antigen tests (see Section 3.1 and Section 3.2). Moreover, since RT-PCR is inaccessible for the majority of laboratories around the world, large scale diagnosis with this method is difficult [61]. Novel, more accurate and sensitive RT-PCR-based methods are under development [62].

### 2.2. Droplet-Digital PCR

An important implementation of the PCR-based methods is the novel droplet-digital PCR (ddPCR) (Figure 1), which is a highly sensitive method for nucleic acid detection and quantification [42,63]. This method is highly sensitive and accurate compared to conventional PCR methods, as it provides a reliable and accurate detection/quantification of pathogenic nucleic acids, including those belonging to SARS-CoV-2 [64,65]. A main advantage of the assay is that it permits the absolute quantification of nucleic acids without using calibration curves. At the same time, ddPCR provides high reliability in detecting low amounts of nucleic acids, by means of single-molecule sensitivity, with lower false-negative results compared to conventional PCR-based methods [42].

Despite having several important differences compared to PCR, the ddPCR workflow is similar to that of a conventional PCR, with potentially identical procedures for (i) primer and fluorescently labeled probes designs (ii) amplification steps/cycles/temperatures. An important step that differentiates the procedure from the conventional PCR is that ddPCR provides the initial random partitioning of the sample into tens of thousands droplets, before amplification. The precise number of droplets varies according to the instrument considered. A single nucleic acid molecule (target molecule) is amplified into each droplet, while droplets are stratified into positive droplets, which contain the target molecule, and negative droplets, which do not contain the target molecule, in accordance with the amplitude of their fluorescence signal [66]. Indeed, each droplet represents an independent amplification event. Lastly, the Poisson statistics provides the absolute copy number quantification of the target molecule [66].

The ddPCR assay was implemented to detect SARS-CoV-2 [67,68]. The method was compared with a conventional RT-PCR and was more efficient in detecting SARS-CoV-2 genome sequences in samples found to be negative with RT-PCR. Moreover, the assay also showed higher sensitivity and accuracy [69]. The ddPCR assay presented a high sensitivity, which was estimated as ~10^−2^ copy/mL, in detecting SARS-CoV-2 nucleic acid isolated from pharyngeal swab samples from COVID-19 convalescing patients [70]. Moreover, the method was also demonstrated to be reliable for COVID-19 patient monitoring by evaluating disease progression [14,71]. Moreover, two additional studies compared ddPCR and traditional qPCR methods for the detection of SARS-CoV-2 RNA [72,73]. In the first study, the ddPCR was performed to the trace detection of exogenous SARS-CoV-2 RNA in wastewater from low-prevalence COVID-19 locations, and relied on two assays named CDC N1 and CDC N2 [72]. The second study tested both ddPCR and qPCR to detect low amounts of SARS-CoV-2 RNA from clinical samples [73]. Both studies underlined that the ddPCR assay is more suitable for determining a low copy number of SARS-CoV-2 RNA, thus suggesting that ddPCR can potentially be used as a highly sensitive and compatible diagnostic method for SARS-CoV-2 RNA detection.

These investigations cumulatively demonstrate the high reliability of ddPCR for clinical purposes. Consistently, two different SARS-CoV-2-based ddPCR approaches were officially approved by the FDA [67]. However, despite the high consistency in detecting and quantifying SARS-CoV-2 nucleic acid with specificity and sensitivity [74], the clinical application of ddPCR seems to be as of yet impractical in diagnostic routine settings of COVID-19. High costs, the need for specific equipment for sample processing, as well as technically difficult experimental procedures are some of the reasons behind its limited use [75]. Indeed, while ddPCR is widespread and accessible, it is still more expensive and labor intensive than RT-PCR, with a longer run time. However, due to its reliability and high sensitivity, the method could be extended to a wider number of subjects with different viral loads and specific clinical pictures, as well to the analysis of environmental samples, including waste water or surface samples, to detect possible virus traces.

### 2.3. Isothermal Amplification-Based Methods

Isothermal amplification-based methods for SARS-CoV-2 detection are represented by isothermal PCR amplification assays [76,77,78,79]. These methods comprise loop mediated isothermal amplification (RT-LAMP), recombinase polymerase amplification (RPA), nicking endonuclease amplification reaction (RT-NEAR), and transcription mediated amplification (RT-TMA) [80,81]. These methodological approaches provide the advantage of shorter turnaround times in comparison to RT-PCR, of approximately one hour in the majority of cases, and most can be employed at the point-of-care (POC) and within limited resource setting.

### 2.4. Clustered Regularly Interspaced Short Palindromic Repeats (CRISPR)-Based Systems

CRISPR-based platforms, also named gene-editing tools (Figure 1) [82,83,84,85], represent new paths for analytical signal amplification with a precision of below single-nucleotide variants [86,87,88,89]. These systems have therefore been rapidly implemented for the detection of viral agents [85,90,91]. These systems are characterized by remarkable sensitivity and specificity alongside a high base resolution and programmability on nucleic acid identification [92]. The most recent form of CRISPR assays for viral detection are a new generation of genome-engineering tools named CRISPR-Cas systems, which use Cas12a and/or Cas13a nucleases. Both CRISPR-Cas12a and -13a assays utilize trans-cleavage of a single-stranded molecule of DNA (Cas12a) or RNA (Cas13a) [68,90]. The detection of viral targets has also been performed with additional CRISPR-Cas platforms such as Cas9 [93,94].

The CRISPR-Cas12a system, named DETECTR (DNA endonuclease- targeted CRISPR trans reporter), is based on a complex named crRNA-Cas12a which recognizes, binds to and cleaves DNA targets, while utilizing FQ-DNA reporters [89]. Broughton and colleagues applied a CRISPR-Cas12a-based system for SARS-CoV-2 identification [95]. The system utilizes an RT-LAMP method for target amplification, while amplicons are recognized by the crRNA-LbCas12a complex, which cuts DNA reporters. The assay detection limit, which is complete in 45 min, has been estimated to be 10 copies/μL [95].

In the CRISPR-Cas13a system, or SHERLOCK (specific high-sensitivity enzymatic reporter unlocking) [87], SARS-CoV-2 nucleic acids are initially amplified by RT-RPA, while amplified DNA is then transcribed to RNA. Subsequently, the complex CRISPR RNA (crRNA)-Cas13a binds to and cleaves the RNA target molecule. At the same time, a fluorescent signal is provided by RNA probes which are conjugated with a fluorescent dye (F) and quencher (Q) pair, which are non-target molecules, i.e., FQ-RNA reporter [87]. The CRISPR-Cas13 system for SARS-CoV-2 identification has been recently designed and provides machine learning algorithms to generate a multiplexed panel comprising 67 different assays to identify the virus [96]. Target molecules are amplified by RT-RPA assay and recognized by the crRNA-LwaCas13 complex. The complex then cuts the RNA reporters. Moreover, a multiplexed CRISPR-Cas13 assay named SHERLOCK-version 2 (SHERLOCKv2) was also recently developed to detect four different RNA targets in a single reaction simultaneously using different Cas13 enzymes [93].

As for other molecular assays, the requirement of isolated and purified nucleic acid amplification to achieve high sensitivity levels can be considered as a relative limitation for CRISPR-Cas systems. Different approaches have therefore been developed to overcome this drawback [97,98,99,100]. For instance, Myhrvold et al. developed a system named Heating Unextracted Diagnostic Samples to Obliterate Nucleases (HUDSON), which is able to identify multiple viral targets from unextracted clinical specimens, including saliva with minimal sample processing [98]. A CRISPR-Cas12-based platform aimed to identify SARS-CoV-2 RNA from saliva was also recently developed by Curti and colleagues [100]. However, this method has a low detection limit, which has been estimated as 10^5^ copies/μL, compared to the ~10 copies/μL determined in SARS-CoV-2 RNA–spiked buffer samples [100]. Lastly, a recent CRISPR-Cas13-based system was developed to identify SARS-CoV-2 directly from collected clinical samples, including saliva and nasopharyngeal swabs, with high efficiency, and thus without nucleic acid isolation [101].

In summary, given their high sensitivity and reliability, CRISPR-Cas systems could potentially be used in the diagnostic screening of SARS-CoV-2 infection across the general population [102].

### 2.5. SARS-CoV-2 Genome Sequencing Methods

Since the beginning of the pandemic, huge efforts have been undertaken to molecularly characterize the SARS-CoV-2 genome sequence [103,104]. Several viral genome sequencing approaches have been applied in order to sequence the various SARS-CoV-2 strains. Considering the various SARS-CoV-2 variants currently identified, which are characterized by a large catalogue of mutations/polymorphisms, the number of sequenced genomes is increasing rapidly. Since the number of SARS-CoV-2 strains is continuously increasing, the WHO published guidelines for the sequencing approaches of the SARS-CoV-2 genome [105].

The currently employed methods for viral genome sequencing can be categorized as follows: (i) metagenomic sequencing, (ii) PCR amplification sequencing and (iii) target enrichment sequencing [104]. Metagenomic sequencing approaches are highly reliable in characterizing pathogen diversity in environmental and clinical samples and for the identification of novel microorganisms [106]. The method isolates the pathogen DNA from a biological/clinical sample, while the library is prepared and sequenced by shotgun sequencing or RNA sequencing (RNA-seq). The main advantages of metagenomic sequencing are the cost-effective sample preparation and the non-necessity of primer or probe design [104]. The characteristic that the clinical samples should have a high pathogen DNA load as well as the relatively low sensitivity can be considered important disadvantages. PCR amplification sequencing-based methods can also be used for genome sequencing. The method is one of the most common sequencing approaches used for enriching viral genomes as it uses primers that are complementary to a known nucleotide sequence [104]. According to the WHO guidelines, complete genome sequencing can be conducted with Ct values of up to 30, while partial genome sequencing can be performed with Ct values in the range of 30–35 [105]. Primer sequences, experimental recommendations, and bioinformatic resources to facilitate SARS-CoV-2 genome sequencing have been developed by the ARTIC network [107]. The network also provides the most widely used primer panels for the SARS-CoV-2 genome sequencing. Three ARTIC network amplicon sets-based primer panels., i.e., Qiagen, and NEBNext ARTIC SARS-CoV-2 Library Prep Kit, CleanPlex SARS-CoV-2 Panel and Paragon genomics, QIAseq SARS-CoV-2 Primer Panel, are also available. Important advantages of the PCR amplification sequencing-based methods include high specificity and sensitivity and good coverage even in conditions of a low pathogen load; these methods are also relatively less expensive compared to other sequencing methods. In contrast, the limited ability to sequence novel/unknown pathogens, the possible establishment of amplification mutations and the fact that PCR reactions are subject to primer mismatch can be considered important disadvantages [104].

Alternative sequencing approaches include target enrichment methods, which have recently been applied to characterize and monitor SARS-CoV-2. Such techniques can be used to sequence whole viral genomes directly from clinical samples without the need for prior PCR amplification or pathogen culture. In this case, the genome of specific pathogens can be enriched via hybrid capture probes which are complementary to a pathogen reference sequence, or to a panel of reference sequences. The characteristic high specificity of these sequencing methods decreases the sequencing costs compared to metagenomic sequencing, thus making target enrichment methods more economically competitive. However, biological sample preparation is expensive, while the method also requires technical expertise. An additional, important limitation is that these methods are unable to sequence genomes of novel, unknown pathogens, therefore requiring well-characterized reference genomes for designing probes [104].

Next-generation sequencing (NGS) has been rapidly applied for research purposes along with conventional sequencing methods [108,109,110], such as direct sequencing [111,112,113], particularly for the identification of novel viral strains [114]. NGS has become essential for the first sequence analysis of the SARS-CoV-2 genome as well as for identifying virus-positive patients at the beginning of the COVID-19 pandemic (Figure 1) [90,115]. NGS platforms therefore provided a method to design a large variety of continuously improved primers and probes for RNA/DNA-based assays, including PCR and implemented assays [46]. Furthermore, the identification of the SARS-CoV-2 sequence allowed for the structural characterization of viral proteins, mechanisms of action and transmission as well as the development of therapeutic approaches [59,116,117].

Overall, metagenomic NGS methods can be subdivided into short-read and long-read approaches [118,119]. Although NGS are increasing in read length, the highest-output platforms produce relatively short read lengths, in the order of 35–600 base pairs per read. The short-read sequencing-based NGS platforms are the most commonly employed NGS platforms in laboratories. In contrast to short-read protocols that read a few hundred nucleotides at a time, long-read sequencing technologies are capable of reading longer lengths in one run, between 5000 and 100,000 base pairs, although much longer reads have also been reported [119]. Long-read NGS is particularly important for genomics/transcriptomics investigations as a complement of short-read-based platforms.

The NGS workflow includes (i) DNA fragmentation and ligation of adapters, also known as library preparation; (ii) DNA fragments amplification (iii) sequencing amplified DNA strands via a sequencing–biosynthesis approach [120]. Since millions of DNA fragments can be simultaneously sequenced, NGS has been broadly exploited for highly complex genomic analyses [121]. Given the large amount of information, in silico analyses are necessary to assemble the DNA fragments by mapping the obtained individual reads to reference genomes. In general, two main genome assembly approaches have been developed, i.e., reference-based (mapping based) assembly and de novo assembly. The first is typically performed if the genome sequence of the target organism is available. The approach provides the alignment of DNA fragments (reads) to a reference genome, while reads are mapped based on the best match and alignment to the reference genome [122]. The majority of pipelines developed for the genome assembly of SARS-CoV-2, such as Viralrecon, V-pipe and SIGNAL provide a reference-based strategy [123]. De novo assemblies are performed without the necessity of a reference genome. They rely upon connecting the DNA fragments to each other using sequence match overlaps [124], thereby leading to the generation of longer sequences referred to as contigs. As a consequence, the de novo assembly approach can be less accurate, computationally exigent and also time consuming compared to reference-based mapping [122]. On the other hand, de novo assemblies are useful when the pathogen is unknown/poorly understood and also when a suitable reference is absent in the database [125]. A recently reported de novo assembly pipeline developed for the genome assembly of SARS-CoV-2 is PipeCoV [123].

Currently, NGS plays a pivotal role in identifying novel SARS-CoV-2 strains and effectively monitoring the viral pandemic spreading worldwide [126]. This novel methodological approach has also been proposed as a diagnostic tool for detecting SARS-CoV-2 in clinical samples [127], being previously exploited for the identification of a large spectrum of pathogens for similar purposes [128]. SARS-CoV-2 identification via NGS is made possible by the direct sequencing of viral RNA, thus bypassing the possible amplification bias associated with PCR-based methods [129,130]. However, targeted sequencing approaches are required in cases of a low SARS-CoV-2 titer in the collected samples [128]. These approaches comprise hybridization-based capture [131,132], amplicon-based methods [133], as well as CRISPR-Cas based enrichment and Single Molecule, Real-Time (SMRT) sequencing [134].

Although NGS platforms are increasingly implemented for clinical application [135], these assays are not exploited for diagnostic routine settings in the current SARS-CoV-2 pandemic. The complexity of the workflow, such as sample/library preparation protocols and the computational data analysis requiring a high level of expertise to operate and analyze the results, are considered as limiting to some degree.

Several helpful public databases such as National Center for Biotechnology Information (NCBI) Genbank [136], GISAID and Virus Pathogen Database and Analysis Resource (ViPR) are currently available for sequence deposition and the analysis of SARS-CoV-2 genome sequences. In particular, NCBI Genbank is an online public database which belong to the larger online platform NCBI SARS-CoV-2 Resources. This platform provides a large catalogue of SARS-CoV-2 genome sequences, annotations and gene records alongside additional biological and clinical information on the COVID-19 pandemic. The main aim of NCBI SARS-CoV-2 Resources is to provide comprehensive molecular and clinical information on SARS-CoV-2 to scientists, bioinformaticians and clinicians. Concerning SARS-CoV-2 sequences, the platform permits the submission of assembled reads of SARS-CoV-2 with FASTA files and source metadata without annotations [136]. Indeed, following sequence submission, which is processed and released into GenBank within about 2 h, the sequence is automatically assessed for quality and annotated with the viral annotation tool Viral Annotation DefineR (VADR) [137]. Established in 2008, GISAID is a global science initiative and primary source which provides free access to the genomic data of a variety of viruses, including SARS-CoV-2. It is considered the largest repository of SARS-CoV-2 sequences worldwide. Indeed, after the outbreak of the COVID-19 pandemic, the majority of the generated and shared SARS-CoV-2 whole-genome sequences were uploaded to GISAID [138]. ViPR is a comprehensive and publicly available database funded by the National Institute of Allergy and Infectious Diseases, which is a component of the NIH [139,140]. The online tool provides several functions, such as search, analysis, visualization and the saving and sharing of data from viral pathogens, including SARS-CoV-2. A variety of web-based analyses, such as multiple alignments, view phylogenetic trees, 3D visualizations and sequence variation determination can also be performed without charge with this online tool [140].

## 3. Immunological Diagnostics for SARS-CoV-2 Infection

Besides molecular techniques [141,142,143,144], additional assays, such as serological testing, have been rapidly developed since the beginning of the COVID-19 pandemic (Figure 1). Molecular tests are of critical importance for COVID-19 diagnosis, as respiratory symptoms including fever, dry cough and breathing difficulties overlap with those of the common cold and flu [68]. In addition, molecular tests should ideally be employed to diagnose viral infection only during its initial phase. For these reasons, it became necessary to use more inexpensive and rapid diagnostic approaches for SARS-CoV-2 antigens and/or anti-SARS-CoV-2 human antibodies (Abs) in blood, salivary, nasal and/or oropharyngeal swabs samples [59]. Immunoassays are able to detect the presence of both viral antigens and anti-viral Abs produced as an immune response to infection [67,145,146]. Molecular and immunological assays can be used to detect currently ongoing or past pathogen infection. In addition, immunological tests also can be used to reduce the occurrence of false-negative results [147,148]. Indeed, both anti-viral Abs and viral antigens are more stable than SARS-CoV-2 nucleic acid, and thus are less prone to degradation during storage/transport procedures.

### 3.1. Antibody-Based Immunoassays

Antibody-based tests can determine the presence and concentration of circulating Abs to SARS-CoV-2 in the plasma/serum/blood in order to evaluate the immunological status of a suspected COVID-19 patient (Figure 1, Table 1) [39].

The Ab response level can potentially vary according to age, gender, as well as the presence of additional comorbidities [149,150]. Immunoglobulin G and M (IgG and IgM) are used as markers for SARS-CoV-2. IgMs are specifically used as indicators of the identification of SARS-CoV-2 early stage infection, while higher IgG levels are identified during late-stage infection or even post-recovery [151,152,153,154]. Obtaining antigens that can be recognized by Abs is the basis for the accurate detection of specific Abs. SARS-CoV-2 antigens used for antibody detection are usually artificial and prepared using genetic engineering technology [155,156]. Considering previous data on SARS-CoV-2, S and N viral proteins would be the main immunogens present among the four structural proteins, i.e., S, E, M, N proteins [152,157]. Okba et al. analyzed the similarity of S and N proteins among human CoVs, such as SARS-CoV-2, SARS-CoV-1 and MERS-CoV, and found that the S1 subunit in the SARS-CoV-2 S protein had the least overlap with other CoVs [152]. Therefore, S1 and N proteins are currently considered to be the most suitable proteins and are used as immune-antigens for COVID-19 serologic tests [68].

The most common antibody-based tests are based on enzyme-linked immunosorbent assay (ELISA) systems [39,63,146]. The ELISA test is a chemiluminescent or fluorescent and colorimetric, microwell plate-based method employed for the identification of a large variety of immunoglobulins by the binding between the designed immune-antigen and the specific target antibody molecule; this interaction leads to a detectable signal [59]. The assay usually uses a 96-well plate which is coated with SARS-CoV-2 immunogenic antigens. Methodologically, patients’ serum, plasma and/or blood are added to the plate wells [158]. In case of a positive signal, SARS-CoV-2 antigens are recognized/bound by the anti-SARS-CoV-2 Abs which are possibly present in the sample [59].

After washing, an enzyme-conjugated secondary Ab (the enzyme is usually horseradish peroxidase) binds to the antigen-Ab complex. Following the addition of the substrate, a color-changing reaction occurs. The color change is a quantitative measure of the amount of Abs present in the clinical sample [159]. The color change is specifically read by a spectrometer, following which the Ab concentration can be calculated with precision [39]. ELISA results, which are currently considered highly specific and sensitive, can be obtained in between 1 and 5 h [59].

The other most commonly used enzyme immunoassay in clinical laboratories is the chemiluminescence immunoassay (CLIA) [67]. The method is chemiluminescent, and can detect and quantify total SARS-CoV-2 Abs or Abs against one of its protein components such as the S and/or N proteins in the sample [160]. SARS-CoV-2 antigens are conjugated with fluorescein isothiocyanate and bound to magnetic particles. Abs in the sample bind to antigens and can be visualized by chemiluminescence using a detection antibody labeled with isoluminol [159]. The main advantages of these methods include their wide dynamic range, high signal intensity, the absence of interfering emissions, high stability of reagents and their conjugates and reduced incubation time [160]. The analytical sensitivity and short turnaround time are considered as advantages of CLIA compared to traditional ELISA tests [67].

Lateral Flow Immunoassay (LFIA) is a rapid immunochromatography method based on antigen–Ab interactions occurring on porous membrane surfaces [59]. LFIA uses gold-tagged SARS-CoV-2 antigens, i.e., S and N proteins, to identify human IgA or IgG and IgM against SARS-CoV-2 [161]. Usually, LFIA requires the use of a few drops of blood [162]. Anti-SARS-CoV-2 human Abs bind to the gold-tagged antigens pre-attached to the membrane [59]. In cases of a positive sample, anti-SARS-CoV-2 Abs bind to the antigens, resulting in a visible band [159]. LFIAs’ advantages include that it is a time-saving, i.e., ~15 min, and straightforward procedure. In addition, LFIAs are easy to perform as they do not require complex devices/protocols [159]. However, LFIA tests present limitations which include, mainly, reduced sensitivity and specificity [163]. A variable performance of the different LFIAs has also been reported [164,165,166]. For these reasons, these methods are currently not considered as a reliable approach for SARS-CoV-2 immunity diagnosis [167]. To address these drawbacks, serological results should be interpreted in conjunction with clinical symptoms, PCR tests and additional laboratory methods/protocols [154]. To increase the accuracy, sensitivity, and detection throughput of LFIA tests, new approaches are currently under development [39]. 

Virus neutralization test (VNT) is considered the gold standard to determine if a patient has active Abs against a virus, while its use for detecting SARS-CoV-2 is limited. In general, the VNT assay provides serial dilutions of plasma and/or serum which are then incubated with the target virus [68]. The sample is subsequently added to viral-susceptible cells and cultured for about 24–36 h. The test results are then measured via microscopy in terms of the cytopathic effect that the virus causes to the cells [168]; neutralizing Abs would block virus replication to allow cells to grow. The VNT is highly valuable in the early phases of an infection, when other commercial assays are unavailable [169]. Despite its diagnostic/research utility, the method requires cell culture facilities and trained personnel [67]. To circumvent the risk of SARS-CoV-2 particles spreading, researchers established the so called pseudovirus-based neutralization assays (PBNAs) using pseudoviruses (PSVs) as harmless surrogates for the SARS-CoV-2 virus [170,171]. PSVs has been used for VNTs with plasma samples from COVID-19-recovered patients [117]. 

### 3.2. Antigen-Based Immunoassays

A growing number of studies are focused on SARS-CoV-2 antigen detection (Table 1) [155]. The antigen-based tests detect SARS-CoV-2 components, mainly S and N proteins, or even the whole viral particle directly in respiratory and blood samples from patients using specific Abs [172]. Different antigen-based immunoassays for the detection of SARS-CoV-2 antigens, such as ELISA and LFIA have been developed and validated [67]. 

Antigen-based assays can be performed on LFIA strips for rapid virus identification or via ELISA tests for an improved sensitivity; for instance, the measurement of 96 samples in parallel [39]. Compared to molecular methods, rapid antigenic tests present a more rapid execution time, of about 15–30 min, easier protocol, require untrained personnel and a lower cost [173]. However, these methods provide a low sensitivity and specificity estimated at 56.2% and 99.5%, respectively [163]. In addition, these methods only allow for the identification of an active SARS-CoV-2 infection [39].

Rapid antigen-based tests for SARS-CoV-2 identification, which target mainly S, N and ORF1 antigens, are broadly required. As a result, a variety of such tests are currently available on the market [174]. Moreover, due to the frequent mutations in S proteins and drastic population change of variants, the target of many rapid antigen test kits is shifting from S proteins to N proteins. The Sofia 2 SARS Antigen Test Kit (or Sofia 2, QUIDEL company) is one of the currently commercially available kits [175]. Sofia 2 comprises a sandwich-type immunofluorescence strip alongside an instrument which detect SARS-CoV-1 and -2 N proteins; the assay is unable to specifically identify SARS-CoV-2 immunoantigens [39]. As an alternative to Abs, strategies based on the systematic evolution of ligands by exponential enrichment (SELEX) are reliable in identifying affinity ligands such as aptamers specific to SARS-CoV-2. Aptamers are nucleic acids that are able to bind molecules or proteins; aptamers are selected with a SELEX strategy starting from a library of nucleic acids [90].

Two others Ag-based immunological assays for detecting SARS-CoV-2 antigens, which were recently approved by FDA, include the BinaxNOW COVID-19 Ag Card and BD Veritor System [81]. The first is based on lateral flow technology, while the BD Veritor System is a chromatographic digital immunoassay. These two Ag-based immunoassays are designed for the rapid identification of SARS-CoV-2 nucleocapsid antigens from samples. The Veritor test has an agreement from 81.8% to 87.5% with the results obtained using PCR-based methods [176]. Another study estimates that the sensitivity of the Abbott BinaxNOW COVID-19 Ag Card is 40,000 to 80,000 copies/swab [177]. Although these assays have only been validated in a few studies, they might have a significant impact in the rapid identification of SARS-CoV-2 [67].

In summary, rapid antigen tests are broadly employed for screening approaches [178,179]. Nevertheless, these tests do not ensure a reliable COVID-19 diagnosis. As a result, antigen-based immunoassay tests require validation with molecular methods [59].

### 3.3. SARS-CoV-2, Eye, Oral and Specific Immune Response

A still unclarified aspect of SARS-CoV-2 infection related to the role of the local immune response against the virus, primarily at the site/s of virus entry. Secretory mucosal IgAs (sIgAs) are in fact known to be essential in controlling a virus entering the body via mucosal surfaces [180]; yet, only a few studies have investigated the potential sIgA response during SARS-CoV-2 infection and its eventual protective role toward the development of severe COVID-19, as most studies have focused on serum antibodies and systemic cell-mediated immunity including innate responses [181]. Of note, sIgA are produced in quantities far exceeding those of all other Ig isotypes combined [182], and IgA presence on mucosal surfaces exposed to infectious pathogens makes them uniquely positioned to intervene in infection establishment and transmission. SARS-CoV-2 may directly interact with both the nasopharynx-associated lymphoid tissue (NALT), including the lacrimal duct and the oral cavity, and with the bronchus-associated lymphoid tissue (BALT); thus searching for the presence of a mucosal immune response in those sites seem important, especially in light of the observation that systemic and local nasopharynx antiviral responses within individuals are poorly correlated, suggesting independent regulation [183], that IgA serum concentration persist longer in saliva than in serum [184], and that IgA neutralization may be associated with protection against reinfection [185]. Additionally, mucosal IgA responses have been demonstrated in infected persons even in the absence of serum antibody responses, suggesting that mucosal responses may play a key role in the early restriction of virus replication at the site of entry [186]. An anti-SARS-CoV-2 mucosal immune response can be essentially detected by specific CE-IVD ELISA assays recognizing the type A antibodies directed against the virus S1 protein (Euroimmun, Lubeck, Germany). Such assays, although semi-quantitative, present high specificity/sensitivity for IgA detection in serum/plasma samples (>95%), and can be used to detect anti-SARS-CoV-2 IgA in saliva and ocular fluids [14,31], after adjusting the protocol for these clinical specimens. Briefly, for oral rinse and tear analysis, the samples are diluted 1:5 in saline, thus allowing for the optimal detection of IgA and differentiation between positive samples and controls, and positivity is expressed following the manufacturer’s instruction, as the ratio (R) between the absorbance (OD 450 nm) value detected in samples and that detected in the calibrator sample provided by the manufacturer. Using this method, anti-SARS-CoV-2 sIgAs were detected in around 34–40% of tear samples [31,32], and in 64.1% of saliva specimens from COVID-19 patients [14]. Notably, the extent of the salivary sIgA response correlated inversely with COVID-19 symptom severity (Spearman r −0.355; 95% CI −0.600 to 0.047; *p* = 0.02), suggesting that a prompt local immune response could control virus replication at the site of entry, preventing the further spread of the virus. Similarly, the anti-SARS-CoV-2 sIgA in the conjunctival fluid was more abundant in asymptomatic as well as paucisymptomatic COVID-19 patients compared to severely symptomatic subjects, although no statistically significant correlation was detected, perhaps due to the low and none-significant number of analyzed subjects [31]. Consistent with these data, recent reports support the hypothesis that mild disease is associated with mucosal-specific sIgA secretion (tears, nasal fluid, saliva), whereas systemic antibody titers, including serum IgA, correlate with severe COVID-19 [187].

Recently, quantitative ELISA protocols were developed and commercialized, recognizing the virus N and S1 proteins and providing a true quantification of the IgA response (RayBiotech, Peachtree Corners, GA, USA) [188], which could be directly measured in saliva or tears and may serve as a marker of the host immune response and as an early diagnostic/prognostic marker.

Moreover, since the sIgA presence may be protective against the development of severe COVID-19 at the lung level, the quantitative assessment of mucosal IgA response by ELISA could also be useful to analyze the development of the mucosal IgA response in vaccinated subjects [189]. Based on preliminary observations, intranasal vaccines for SARS-CoV-2 that are able to elicit both systemic and mucosal immunity are being investigated [190,191].

## 4. Conclusions

COVID-19 emerged onto the world stage in late 2019 in China and quickly spread across the globe causing, to date, over 423 million cases and 5.88 million deaths. Over these last 2 years, enormous progress has been made in the implementation of techniques aimed at exploring molecular diagnostics. Of particular importance are the new and highly sensitive PCR and RT PCR methods, the most recent CRISPR-based systems which explore both ddPCR amplification assays and isothermal PCR, as well as NGS which has been applied for research purposes along with conventional sequencing methods, including direct sequencing [112,113,192], especially in the context of identifying new viral strains [193] or variants of SARS-CoV-2.

To this end, more rapid and low-cost diagnostic strategies have been adopted to either detect viral antigens, i.e., antigen-based immunoassays or human anti-SARS-CoV-2 antibodies, i.e., antibody-based immunoassays, in nasal or oropharyngeal swabs, as well as in blood or saliva samples. These techniques have helped to partially overcome the limitations of molecular methods that are also affected by the sample type and the timing of infection, with their effectiveness being optimal primarily in the early stages of viral infection. Furthermore, due to the nature of PCR, the target sequence that is amplified is one of the most important design points that must be addressed in order to ensure accurate diagnosis, especially when dealing with new variants and mutations of the virus. In this context, there is a lack of information about the presence of SARS-CoV-2 or its genomic material in both the oral cavity and the eye, as well as in other viral targets expressed in the ACE2 receptor. Thus, the role of the local immune response against the virus, primarily at the site/s of virus entry is crucial and needs to be explored. Mucosal sIgAs are essential in controlling several viruses entering the body via mucosal surfaces, although few studies have investigated the presence of an sIgA response during SARS-CoV-2 infection and its eventual protective role toward the development of severe COVID-19, especially at the lung level. From preliminary experiments, anti-SARS-CoV-2 sIgAs in the conjunctival fluid were found to be more abundant in either asymptomatic or pauci-symptomatic COVID-19 patients compared to severely symptomatic subjects, although no statistically significant correlation was detected, perhaps due to the low number of analyzed subjects [31]. Consistent with these data, the hypothesis that mild disease is associated with mucosal-specific sIgA secretion (tears, nasal fluid, saliva), whereas systemic antibody titers, including serum IgA, rather correlate with severe COVID-19, becomes more and more consistent. The mucosal sIgA response evoked by SARS-CoV-2 may therefore play a crucial role in virus control and its clearance, and could thus serve as an early and potential new marker of the host immune response, with potential prognostic value also in patient monitoring and in the prediction of protection in vaccinated patients.

## Figures and Tables

**Table 1:** Immunological diagnostics for SARS-CoV-2 infection.

Assay	Sample Type	Target Molecules	Detection Time (after Symptoms)
Enzyme-Linked Immunosorbent Assay (ELISA)	Serum	IgA	First Week
		IgG	From Week 2
		IgM	From Week 2 to Week 6
Chemiluminescence Immunoassay (CLIA)	Serum	IgG	From Week 2
		IgM	From Week 2 to Week 6
Lateral Flow Immunoassay (LFIA)	Whole blood	IgA/IgG and	First Weeks
		IgM	From Week 2 to Week 6
Virus Neutralization Test (VNT)	Serum	Neutralizing antibodies	After recovery
Lateral Flow Immunoassay (LFIA)	NPS/OPS	S and N	From Week 1 to Week 4
	Serum		
Enzyme-Linked Immunosorbent Assay (ELISA)	NPS/OPS	S, N and ORF1	From Week 1 to Week 4
	Serum		
Immunochromatographic Assay (ICA)	NPS/OPS	N	From Week 1 to Week 4

N, Nucleocapsid protein; S, Spike protein; NPS, Nasopharyngeal swab; OPS, Oropharyngeal swab.

## Data Availability

Not applicable.
